# Maternal deaths in Pakistan: intersection of gender, caste, and social exclusion

**DOI:** 10.1186/1472-698X-11-S2-S4

**Published:** 2011-11-08

**Authors:** Zubia Mumtaz, Sarah Salway, Laura Shanner, Afshan Bhatti, Lory Laing

**Affiliations:** 1 University of Alberta, School of Public Health, Edmonton, Alberta, Canada; 2 Sheffield Hallam University, City Campus, Howard Street, Sheffield, UK

## Abstract

**Background:**

A key aim of countries with high maternal mortality rates is to increase availability of competent maternal health care during pregnancy and childbirth. Yet, despite significant investment, countries with the highest burdens have not reduced their rates to the expected levels. We argue, taking Pakistan as a case study, that improving physical availability of services is necessary but not sufficient for reducing maternal mortality because gender inequities interact with caste and poverty to socially exclude certain groups of women from health services that are otherwise physically available.

**Methods:**

Using a critical ethnographic approach, two case studies of women who died during childbirth were pieced together from information gathered during the first six months of fieldwork in a village in Northern Punjab, Pakistan.

**Findings:**

Shida did not receive the necessary medical care because her heavily indebted family could not afford it. Zainab, a victim of domestic violence, did not receive any medical care because her martial family could not afford it, nor did they think she deserved it. Both women belonged to lower caste households, which are materially poor households and socially constructed as inferior.

**Conclusions:**

The stories of Shida and Zainab illustrate how a rigidly structured caste hierarchy, the gendered devaluing of females, and the reinforced lack of control that many impoverished women experience conspire to keep women from lifesaving health services that are physically available and should be at their disposal.

## Background

Complications of pregnancy and childbirth remain the leading cause of death and disability for childbearing women in many low and middle income countries. Of the estimated 529,000 maternal deaths globally each year, 99% take place in the developing world [1]. over half in just six countries [2]. The obvious remedy is to provide competent maternal health care during pregnancy and childbirth [3-5]. Contemporary policy espoused by the World Health Organization and the United Nations Population Fund (UNFPA) promotes this medicalized approach, which subsequently has grounded the national maternal health policies of many developing countries, including Pakistan [6,7].

However, despite significant international investment under the Safe Motherhood Initiative and strategies to achieve Millennium Development Goal No. 5, countries with the highest burden of maternal mortality are failing to reduce their mortality rates [8-10]. This suggests the medicalized approach may be necessary but is insufficient to achieve the desired reductions in maternal mortality. A large, but parallel body of public health and development literature demonstrates that women’s use of reproductive health services is determined not only by the physical availability of such services, but also by gendered inequities that shape women’s access to education, ability to travel, financial and social resources, and decision-making authority in key aspects of their lives [11-13]. These gendered inequities are further intersected by class and caste-based hierarchies, resulting in large differentials in access to services between rich and poor women within countries. Consequently, maternal health care is one of the most inequitably distributed health resource even within poor countries. A recent analysis of Demographic Health Survey data from 33 low and middle income countries shows that the odds of having a skilled birth attendant at delivery for women in the poorest quintile are 94% lower than for women in the highest wealth quintile [14]. Women with primary education are five times more likely to have skilled birth attendance compared to women with no formal education [15-17].

Generally lacking from this body of literature, however, is insight into the underlying political and social dynamics that create and reinforce these inequities in access to care. An understanding of the root causes, and the political and social dynamics that reinforce these inequities remains a critical gap in our knowledge. In particular, we know very little about the ways in which economic class and gender influence healthcare policy formation, resource allocation, the design and delivery of maternal health services, and disadvantaged women’s access to health services. Material poverty alone does not explain the large disparities in access to maternal health care between the rich and poor [18]. Poverty is relational and embedded within power hierarchies [19,20]. We therefore drew upon the concepts of social exclusion and adverse incorporation [21-25] to understand how power hierarchies operating at family, community and state levels conspire to prevent women from accessing timely maternity care despite the physical accessibility of maternity services [19,20]. Three frameworks grounded our inquiry: the Monopoly paradigm describes cultural distinctions such as class, political power, and economic priorities as perpetuating inequality and domination [26]. Kabeer expands this approach to include perceptions of social identity related to caste membership [27], and the “social relations” analytical framework addresses gender [28].

The current paper presents early findings from an innovative research study in rural Punjab, Pakistan that aimed to address these issues. Pakistan’s maternal mortality rate stands at 297/100,000 live births, among the six worst rates in the world. Pakistan is also characterized by deeply entrenched gender inequities [29] and vast inequities in access to maternal health care services, illustrated by the fact that while 77% of women in the highest wealth quintile report receiving skilled care at delivery, this figures is just 16% in the lowest wealth quintile [30]. Two broad questions were addressed by the research 1) how are the axes of social exclusion constructed, mobilized and experienced in Pakistan, particularly in relation to class and gender divisions? 2) how do these forces affect access to maternal health services?

Through the case histories of two women who died shortly after childbirth that we present below, we can begin to see how gender and socio-economic hierarchies, underpinned by societal discourses that sytematically devalue particular sub-groups and legitimize discriminatory behaviors, and shaped by wider ideologies and economic structures, contribute to tragic outcomes in a location where maternal health services are physically available.

### Contextual background

Here we present a brief overview of the social structure of the village for we argue that not only does it underpin chronic poverty, it also determines the nature of social relations and modulates women’s access to maternal health services. Village Gaind-Pind is a well-defined settlement that is connected by an all-weather road to the main highway of the country. Although agriculture and livestock rearing are key economic activities, the village is situated in the ‘martial belt’ of northern Punjab from which the Pakistan Army has traditionally drawn its soldiers.

Consequently, a large proportion of men work in the army, police and the government. There is a small oil and gas development company recently established in the village, but it has minimum interaction with the villagers. There are three primary schools in the village, one for boys and girls each and one co-educational. A range of maternal health care services are available in this community, including three public sector first-level care facilities that provide round the clock obstetric services, and a physician who provides basic obstetric care in a private practice. Within an hour’s drive are a popular birth attendant of uncertain training, and a large district hospital; two teaching hospitals exist about four hours’ drive away.

## Methods

Our study adopted a critical ethnographic approach, which allowed us to synthesize the traditional ethnographic focus on subjective meanings and beliefs of the respondents with the insights gained from a broader historical and structural analysis [31]. The ethnographic method, with its emphasis on immersion in the research setting enabled us to develop a “thick description” of the cultural understandings of caste, class and gender, and to elicit subtleties of power and resource distribution [32].

The findings presented in this report are based on data generated in the first six months of field work in the village of Gaind-Pind, Northern Punjab, Pakistan (note that the name of the village has been changed). Data were collected in four overlapping phases by a team of four researchers (three women, one man). The primary investigator (PI) (Zubia Mumtaz) lived in the village for three months and maintained regular contact with the team in the field. In Phase One, four social mapping exercises and informal home visits enabled us to introduce ourselves to the villagers and familiarize ourselves with village life. Phase Two consisted primarily of informal interviews and observation/participation in the daily life of the village. Investigators digitally recorded detailed, twice-daily summaries of their activities, observations and conversations with villagers, supplemented by their personal impressions of the settings and behaviors in order to provide commentary on the context and meaning of narratives and not merely their face-value content. Phase Three focused attention on pregnant women and members of the lowest castes in the village, continuing to use mixed methods. Phase Four focused on constructing detailed case studies of the five maternal deaths that had occurred in the village within the past five years (one during the field work period). Interviews were conducted in Potohari and Urdu, and digitally recorded if permission was granted. All recordings were translated and transcribed by native Potohari and Urdu speakers under close supervision of the PI (ZM). The transcripts were open coded manually and recategorized. The two case studies we present below were pieced together from information gathered from different sources and the data was searched for patterns and insights regarding the events surrounding the two maternal deaths and their inter-relationships with gender and socio-economic class.

## Findings

### The stories of Shida and Zainab

#### Shida

Shida was a Kammi woman (see below for an explanation of the caste system) who had received no antenatal care and planned on delivering at home. When she went into labour, the baby was in a transverse lie. After two days of labor under attendance of two traditional birth attendants, Shida was taken to a private hospital in a small town a 45-minute drive away. A C-section was performed, but the baby died. On day 41 postpartum, Shida complained of pain in her legs and her health deteriorated rapidly. The doctors of a small nearby hospital referred her to a major teaching hospital, but the family decided they could no longer afford her care and let Shida die. Shida, along with her husband, mother-in-law (also her father’s sister) and parents worked and lived at a bhatta, which is a brick making kiln. Bhatta workers are best described as bonded labor. A family enters this arrangement when the family head is forced to take a loan for whatever reason, which must then be repaid with interest. Workers are paid Rs 300 (US$2.50) per 1000 bricks; a family of four adults can make about 2000 bricks per day, earning the equivalent of US$5.00. In order to keep up with debt payments, men, women, old men and even children as young ten years of age need to work under horrendous conditions from sunrise to sunset (4 am to 7 pm), with only a break for lunch.

When Shida required medical care during childbirth and later, her family had no financial or social resources. The medical and funeral expenses for Shida and her baby were borrowed from the bhatta owner, adding to the family’s never-ending debt.

#### Zainab

Zainab, aged 20, also belonged to the Kammi caste, although she was married into a very poor Mianne caste, which is just slightly above the Kammis in status. She lived in an extended marital family outside of her own biradari that included her husband Salim, his mother, his brother, and his sister Ayesha. Approximately six months into the pregnancy, Ayesha kicked Zainab in the abdomen and threw her out of the house. A few hours later, Zainab delivered twin girls who died soon afterwards. Zainab died the next morning, eight hours after the delivery. According to Salim’s mother, Zainab was pale, sweating and limp, and she “just slipped away.” Although the marital family denied there had been any vaginal bleeding, the neighbors said the bed was soaked with blood when Zainab’s body was taken away.

At no stage was Zainab taken to a health facility; no healthcare provider other than a minimally--trained village-based lady health worker was consulted for childbirth, and no one was called between the delivery and Zainab’s death. The family never directly said that they did not seek medical care; we had to probe carefully to confirm that no one was consulted, as the family members were very reticent about discussing these choices. Their narratives were primarily centered on questioning why Zainab died when there was ‘nothing wrong with her’. They were also reluctant to discuss Ayesha’s violence and the role it played in the deaths of Zainab and her babies. Their narratives were devoid of any self-questioning, although the reticence about discussing key events suggests they were aware of it. The neighbors were more forthcoming about injustices meted out to Zainab, primarily in the context of *sasural-bahu* (marital family-daughter-in-law) relations but even they did not make the links between the beating and subsequent death. A maternal death inquiry too did not identify the prior violence.

Clearly, seeking medical care for Zainab would have incurred financial costs that were not considered justified for a number of reasons. Her husband does not work and never provided for Zainab. Her natal family was also very poor. Since this was an exogamous marriage, her natal family could not advocate on her behalf. All these factors located Zainab in a very weak position structurally and therefore extremely vulnerable to harassment by her marital family.

### The social order of the village

Our research revealed the existence of a hierarchal caste system that underpinned chronic poverty and provided some insight into the underlying social and economic dynamics that prevented Shida and Zainab from accessing care that was otherwise physically available. The pivotal social institution of our field site is a kin-group called a biradari. Consisting of a group of households linked by blood, it also constitutes a social, economic and political unit of the village. Members of a biradari adhere quite closely to the basic rules set forth by the rules governing the kinship group. Nonetheless, the *biradaris* are not necessarily harmonious structures; loyalties, solidarity, and animosity can all exist within the same biradari at different times. The close relationships provide the basis for mutual support and cousins are natural allies against any external threat, but in the absence of that, rivalry over the common grandfather’s land provides the basis for bitter, sometimes violent, conflict.

The second level of the social order is the *zaat* or caste system. Similar to the Indian caste system, the *zaat* system is hierarchal, with some blurring at the borders. There are five main zaats in the village with the Chaudhrys (also called Warsis because they are the inheritors of wealth) and Rajas at the top, followed by the Mirzas, the Miannie and finally the Kammis. Pakistan is a context where landownership equates to economic security, power and prestige. Not only do the higher caste Chaudhrys and Rajas own large tracts of land with associated power, they are understood to be ‘*khandaani*’, a term connoting high moral standards, that their word means something; they never beg.

At the bottom of the *zaat* hierarchy are the Kammis. Our preliminary data suggests members of the Kammi *zaat* are socially constructed as inferior. The term Kammi is used to describe this *zaat* because they are, according to the higher caste, ‘*kaam karne wale*’ (those who work). A more nuanced analysis, however, suggests it is the root of the term ‘*kam*’ that is the real descriptor of the social construction of this *zaat*. The word ‘*kam*’ translates into ‘less’ and refers to the widely held belief that this caste has a lower level of ‘*zameer*’ (conscience, virtue or moral character) than people of high castes. The higher castes believe that Kammis, as a group, cannot be relied upon; their word means nothing, they are cowards, they beg. They are quick to point out that they do not consider the Kammis to lack morals because they are the workers, but that the trait of good character is just inherently absent. One insightful informant suggested that the Kammis’ lower *zameer* has deliberately been cultivated by breaking down these people’s sense of self in order to enslave them economically. They are made to sit on the floor, while the higher castes sit on beds; they are not paid for the work they perform, but are forced to ask for what is their due, and then they are accused of being lowly beggars.

The caste system determines the kind of work a person does and the opportunities available and from there economic security and social power. In the economic structure of the village, Kammi families traditionally provide socially ascribed low status tasks such as construction (*mistri*), shoemakers (*mochi*) butchers (*kasai*) barbers (*nai*). They do their assigned duties all year round and are paid in-kind, usually wheat. This system, known as the ‘*seph*’ is inherently abusive as the amount of wheat given varies according to the whims of the higher caste landowners (payment all year long is usually a measure of grain at harvest time, but may be clothing or other items). They are invariably landless and are ‘given’ a small piece of land that they will never own to build a home. This leaves them vulnerable as they can be evicted according to the whims of the landlord. Despite the rigidity of the system, some Kammi families have tried to break away from this sephi system and work for cash, usually as day labourers doing manual work. There is one Kammi family (out of about 20) that has acquired an education and one member works as a medical technician. Nonetheless, chronic, intergenerational poverty is a defining element of this *zaat*.

Our research also generated a detailed understanding of the gender order of this society. Gender roles are sharply demarcated: men are socially constructed as providers of both economic resources and social identity, while women are their dependants. Marriages are usually endogamous, arranged within the biradari. Although brides move to live with their marital families, a woman’s mother, aunts and others can exert protective authority within the *biradari*’*s* structured relationships to ensure her health needs are met. Marrying outside of one’s biradari can leave a woman potentially vulnerable to abuse by unrelated in-laws, and without social support or advocates if she needs help, particularly if the natal family is also poor.

## Discussion

The objective of this paper was to understand the underlying contextual factors that explain why some women experience adverse maternal outcomes, while others do not. We argue poor maternal health and lack of access to maternal health care services is a social phenomena and a form of deprivation. Despite the different details of caste, family dynamics and specific circumstances, the stories of Shida and Zainab illustrate how rigidly structured economic hierarchy, the gendered devaluing of females, and the reinforced lack of control that many impoverished pregnant women experience, conspire to keep them from lifesaving health services that are physically close by and should be at their disposal.

Both women belonged to the lowest castes and were impoverished. Poverty underlay the decision to let Shida die and poverty was the key reason no health care was sought for Zainab despite physical availability of services. Our data show that poverty is not merely inherited or accidental, but is closely interwoven with the caste system. Indeed, of the five maternal deaths that occurred in the five years prior to our fieldwork in the village, four were Kammis. The data also shows that the Kammis are systematically denied access to social and economic resources (land ownership, cash paying jobs, and education opportunities) and the possibility of upward social mobility. However, they are not completely excluded from the structures of society, rather by performing essential work that others refuse to do, the lowest castes are adversely incorporated into the local economy that would collapse without their efforts. The indentured servitude of the *bhattas* is a clear example of how rigidly adverse incorporation can be enforced. Gender inequities further interacted with caste and poverty to kill the two women. Domestic violence is a gendered phenomenon and violence particularly during pregnancy is well documented [33,34]. It serves to perpetuate patriarchal power and is sustained by a culture of silence and denial of the seriousness of the health consequences of abuse [35]. None of the respondents, the marital family, the neighbors, not even the health care providers who conducted the Zainab’s maternal death inquiry made the possible link between the violence and subsequent death.

The sharply demarcated gendered roles in which men are socially constructed as providers of economic resources means that women may become particularly vulnerable if their husbands are unable to provide. Poverty and gender values that perceive women as not “worth” spending money on for medical care [36], in this case maternal health care, means that women whose husbands are unable to provide are doubly vulnerable.

## Canadian-low and middle income country research partnership

This research began as a collaborative project involving the University of Alberta, Canada; the Health Services Academy, Pakistan, and Sheffield Hallam University, UK. Researchers at University of Alberta and Sheffield Hallam University conceptualised the research with support from the local partners in Pakistan. The PI had a joint appointment at the University of Alberta and the Health Services Academy. However, by the time research funding was secured, a change in management at the Health Services Academy and the loss of one local co-investigator who left to pursue her PhD studies meant that the originally planned institutional collaboration could not be actualized to the degree planned. Module 1 of the research project was therefore conducted independent of the local partner. The PI – a person of Pakistani origin - has extensive knowledge and connections on the ground; it is possible that researchers without such extensive local knowledge and relationships may well have had their project collapse under such circumstances. A key lesson learned from this was the importance of individual researchers in maintaining institutional partnerships.

Knowledge translation was a key objective of this research. In the absence of local partner involvement, the PI (ZM) has developed direct and ongoing engagement with policymakers in the Pakistan government, specifically the Maternal and Neonatal Child Health program and bilateral aid agencies (DFID). Acknowledging that there is an inadequate understanding of the deeply rooted multi-dimensional inequities embedded within the societal structures and the role they may play in the high maternal mortality rates, the policymakers appreciate the research as addressing an important gap in knowledge. Nonetheless, personal one-to-one engagement with key stakeholders suggests that the sensitive and contentious nature of the topic mean that discussions involving gender, caste and related social distinctions will remain uncomfortable. Despite the increasing awareness, health policymakers feel that it is difficult for them to address the deep-seated social and economic inequalities that are reinforced by multiple hierarchies.

We conclude that until the complex, underlying dynamics that determine healthcare access are better understood, programs aimed primarily at expanding health services are likely to continue to fail to address inequities and to reduce the level of maternal mortality. The findings of this research have relevance to global health leaders and to maternal health advocates in particular, for they demonstrate that a purely health systems strengthening approach to reducing maternal mortality is inadequate. It is equally important to understand and address gender, caste-and class-based inequities.

## List of abbreviations used

DFID: UK Department for International Development; MDG: Millennium Development Goal; PI: Primary Investigator

## Competing interests

The authors declare that we have no competing interests.

## Authors’ contributions

ZM was PI on the project, leading the overall design and execution of the work. She analyzed the data and wrote the manuscript. SS contributed to study design and provided critical input in the manuscript. AB collected the anthropological data and provided critical input in data analysis. LS and LL helped draft and provide input in the manuscript respectively. All authors read and approved the final manuscript.
